# Isolation of a Human Anti-HIV gp41 Membrane Proximal Region Neutralizing Antibody by Antigen-Specific Single B Cell Sorting

**DOI:** 10.1371/journal.pone.0023532

**Published:** 2011-09-30

**Authors:** Lynn Morris, Xi Chen, Munir Alam, Georgia Tomaras, Ruijun Zhang, Dawn J. Marshall, Bing Chen, Robert Parks, Andrew Foulger, Frederick Jaeger, Michele Donathan, Mira Bilska, Elin S. Gray, Salim S. Abdool Karim, Thomas B. Kepler, John Whitesides, David Montefiori, M. Anthony Moody, Hua-Xin Liao, Barton F. Haynes

**Affiliations:** 1 Duke Human Vaccine Institute and Departments of Medicine, Surgery and Immunology, Duke University School of Medicine, Durham, North Carolina, United States of America; 2 National Institute for Communicable Diseases, Johannesburg, South Africa; 3 Center for AIDS Program of Research in South Africa (CAPRISA), University of KwaZulu-Natal, Durban, South Africa; 4 Laboratory of Molecular Medicine, Children's Hospital, Harvard Medical School, Boston, Massachusetts, United States of America; University of California San Francisco, United States of America

## Abstract

Broadly neutralizing antibodies are not commonly produced in HIV-1 infected individuals nor by experimental HIV-1 vaccines. When these antibodies do occur, it is important to be able to isolate and characterize them to provide clues for vaccine design. CAP206 is a South African subtype C HIV-1-infected individual previously shown to have broadly neutralizing plasma antibodies targeting the envelope gp41 distal membrane proximal external region (MPER). We have now used a fluoresceinated peptide tetramer antigen with specific cell sorting to isolate a human neutralizing monoclonal antibody (mAb) against the HIV-1 envelope gp41 MPER. The isolated recombinant mAb, CAP206-CH12, utilized a portion of the distal MPER (HXB2 amino acid residues, 673–680) and neutralized a subset of HIV-1 pseudoviruses sensitive to CAP206 plasma antibodies. Interestingly, this mAb was polyreactive and used the same germ-line variable heavy (V_H_1-69) and variable kappa light chain (V_K_3-20) gene families as the prototype broadly neutralizing anti-MPER mAb, 4E10 (residues 672–680). These data indicate that there are multiple immunogenic targets in the C-terminus of the MPER of HIV-1 gp41 envelope and suggests that gp41 neutralizing epitopes may interact with a restricted set of naive B cells during HIV-1 infection.

## Introduction

The isolation of new anti-HIV-1 envelope neutralizing human monoclonal antibodies (mAbs) is a high priority since they may identify potential targets for vaccine design. Until recently only a handful of such mAbs were available, and these were isolated either through traditional EBV-transformation or phage-display libraries [1], [2], [3], [4]. Newer state-of-the art technologies utilizing single cell sorting of antigen-specific memory B cells together with PCR amplification of immunoglobulin gene (Ig) fragments have produced additional antibodies including VRC01, a potent new anti-CD4 binding site (CD4bs) mAb [5], [6]. Furthermore, high-throughput neutralization screening of short-term memory B cell cultures yielded PG9 and PG16 mAbs, which are broadly cross-reactive and define a new target on the gp120 envelope glycoprotein [7].

The membrane proximal external region (MPER) in gp41 represents an important target for anti-HIV-1 neutralizing antibodies [8]. This highly conserved stretch of ∼23 amino acids in gp41 proximal to the viral membrane is required for viral infectivity. The broadly neutralizing antibody 2F5 binds amino acid residues 663–667 at the MPER N-terminus with the tripeptide motif _664_DKW_666_ essential for its recognition [9]. MPER mAb 4E10 shows greater breadth and binds residues within the C-terminus with amino acids W_672_, F_673_ and W_680_ critical for binding [9]. Both antibodies have long CDRH3 regions with hydrophobic CDRH3 loops that confer lipid polyreactivity [10]. This enables the antibodies to bind first to virion lipids, which optimizes binding to the gp41 intermediate epitope that is transiently exposed during virion induced cell fusion [11]. A third, less potent mAb Z13e1, overlaps the 4E10 epitope spanning residues 668–677 and makes contact with N_671_ and D_674_ [12]. While anti-MPER antibodies have been detected in plasma of approximately one third of HIV-infected individuals, using chimeric viruses with HIV-1 MPER grafted into a SIV or an HIV-2 envelope glycoprotein, antibodies with 2F5 and 4E10 specificity are extremely rare [13], [14]. The neutralizing capacity of antibodies targeting other epitopes within the MPER is largely unknown.

We recently described anti-MPER antibodies in a chronically infected subtype C infected individual (CAP206) that were responsible for plasma neutralization breadth, and were targeted to the distal MPER centered around D_674_ [15]. Here we describe isolation of a novel anti-MPER neutralizing mAb from this individual, CAP206-CH12; through the amplification of Ig gene fragments from single memory B cells sorted using a fluorescently-labeled MPER-peptide tetramer. This mAb overlapped the 4E10 and Z13e1 epitopes and neutralized a subset of viruses sensitive to plasma antibodies. CAP206-CH12 used the same V_H_ and V_k_ Ig gene families as the 4E10 mAb and its CDRH3 sequence showed strong similarities with that of Z13e1 as a result of shared J gene usage [16]. These data suggest the possibility of convergent evolution among HIV-1 gp41 MPER mAbs.

## Results

### CAP206 Plasma Reactivity and Labeling of MPER-Reactive Memory B Cells

We have previously identified an HIV-1-infected individual from the CAPRISA 002 acute infection cohort in Durban, South Africa who developed broadly cross-reactive neutralizing antibodies targeting the MPER [15]. This was shown by depleting neutralizing activity from plasma using MPR.03 peptide-coated beads. Of the 44 viruses tested, 50% were neutralized by CAP206 plasma of which 68% were confirmed to be neutralized via anti-MPER antibodies [15].

**Figure 1:**
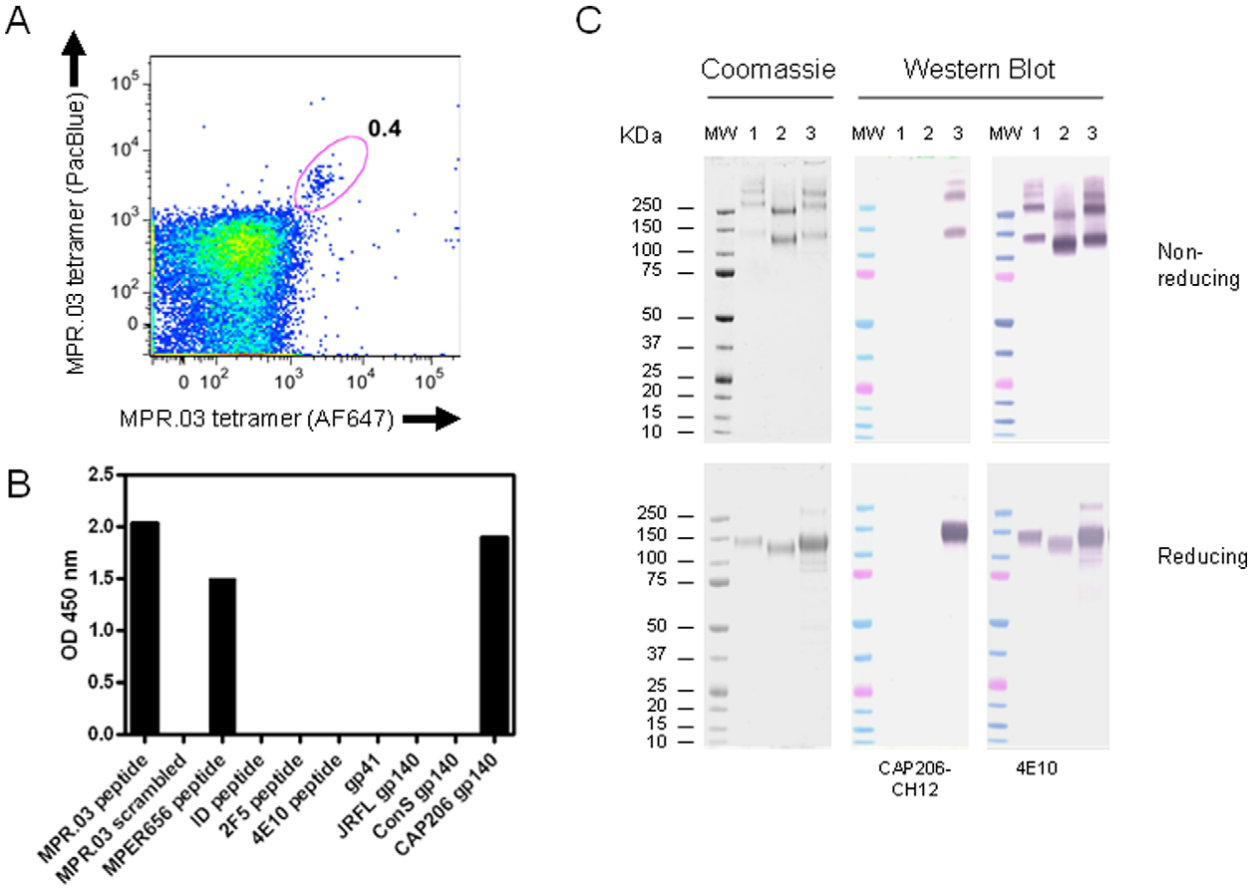
Isolation of the MPR.03-specific mAb CAP206-CH12. (A) Flow cytometric plot of CD19+/CD27+ memory B cells from CAP206 stained with labeled MPR.03 tetramers. Circled cells represent double-positive memory B cells that were single-cell sorted into 96-well plates for Ig gene amplification and expression. B cells from HIV negative individuals did not stain with MPR.03 tetramers (not shown). (B) ELISA data showing binding of CAP206-CH12 to MPR.03 peptide as well as MPER656 peptide. A scrambled MPR.03 peptide was negative as were peptides for the gp41 immunodominant (ID) region (SP400), 2F5 epitope (SP62 peptide) and 4E10 epitope. There was also no binding to JR-FL gp140, ConS gp140 or MN trimeric gp41. Data from a single experiment representative of at least 3 is shown. (C) Binding of CAP206-CH12 to gp140 envelope proteins in Western Blot. HIV-1 envelope proteins JR-FL gp140 (1), ConS gp140 (2) and the autologous protein, CAP206.B5.140C (3) were run under non-reducing and reducing conditions. Western blots were stained with CAP206-CH12 at 2 µg/ml or with the 4E10 mAb as a positive control. Bound mAb was detected using AP-labelled goat anti-human IgG.

The ability to deplete specific antibodies from the plasma of CAP206 using an MPER peptide suggested that it may be possible to label and sort memory B cells producing these antibodies. We therefore designed a tetramer using MPR.03 monomer peptide which was biotinylated and reacted with streptavidin to yield a tetramer with 4 MPER epitopes for B cell surface Ig cross-linking [17]. To decrease the overall labeling background, MPR.03 tetramers were labeled with either AF647 or PacBlue and used to stain PBMC from CAP206 collected at 28 months post-infection. Memory B cells (CD19^+^, CD27^+^) that were dual stained with both MPR.03-AF647 and MPR.03-PacBlue were sorted into individual wells of a 96 well plate (Figure 1A). The frequency of tetramer-specific B cells was approximately 0.4% of memory B cells. Given that memory B cells constituted ∼1–2% of this sample we estimated that the peptide-binding B cells represented less than 1 in 10,000 of total PBMC.

### Isolation of CAP206-CH12, an HIV-1 MPER-Reactive MAb

Single cell PCR amplification and transient expression of Ig genes of sorted B cells yielded an IgG1 mAb, CAP206-CH12 that reacted strongly with the MPR.03 peptide but not with a scrambled version in an ELISA (Figure 1B). It also bound the MPER656 peptide which differed from MPR.03 at position 674 (N in MPER656 and D in MPR.03). It did not bind to a peptide from the gp41 immunodominant region (ID, SP400) or peptides that bound the 2F5 and 4E10. This mAb also did not react with trimeric MN gp41 protein, JR-FL gp140 or the group M consensus Env protein (ConS gp140). CAP206-CH12 also failed to recognize these proteins in Western blots done under reducing and non-reducing conditions (Figure 1C), despite JR-FL and ConS sharing an almost identical sequence to the MPR.03 peptide (JR-FL differs by a single amino acid, 677K). In contrast, CAP206-CH12 bound well to the autologous gp140 protein by ELISA and Western blot.

Remarkably, mAb CAP206-CH12 used the same heavy and light chain gene families as the 4E10 mAb, namely V_H_1-69 and V_K_3-20 and the same J_H_ family as another anti-MPER mAb, Z13e1 (Table 1). It had the shortest CDRH3 (17 amino acids) and the longest CDRL3 (11 amino acids) of the three antibodies. The mutation rate in the heavy chain was similar to 4E10 but it had the highest number of light chain mutations. The CDRH3 of CAP206-CH12 had four tyrosines similar to Z13e1 as a result of the shared J6*03 allele. However, all 3 antibodies were genetically distinct (see Figure S1 for amino acid alignments and nucleotide sequences).

**Table 1:** CAP206-CH12 germ-line gene families compared to 4E10 and Z13e1.*

Antibody ID	V_H_			J_H_	VL		
	Family	CDR3 length	% mutated	Family	Family	CDR3 length	% mutated
CAP206-CH12	1∼69*04	17	11.9	6*03	3∼20*01	11	5.2
4E10	1∼69*10	20	12.6	1*01	3∼20*01	9	7
Z13e1	4∼59*03	19	17	6*03	3∼11*01	9	3.5

*using SoDA algorithm.

In addition to CAP206-CH12, a second functional antibody with a V_H_3-7 heavy chain was isolated but it failed to bind the MPR.03 peptide. A further four heavy chain genes were sequenced, but these were either non-functional or paired light chains could not be amplified. One of these also used V_H_3-7 and unlikely to be specific. However, the other three used the V_H_1-69 heavy chain genes with CDRH3 regions between 11–18 amino acids, similar to CAP206, suggesting that additional MPER-specific mAbs exist. Attempts to repair these or to pair them with the CAP206-CH12 light chain were unsuccessful.

### Characterization of Binding Sites and Affinity of CAP206-CH12

**Figure 2:**
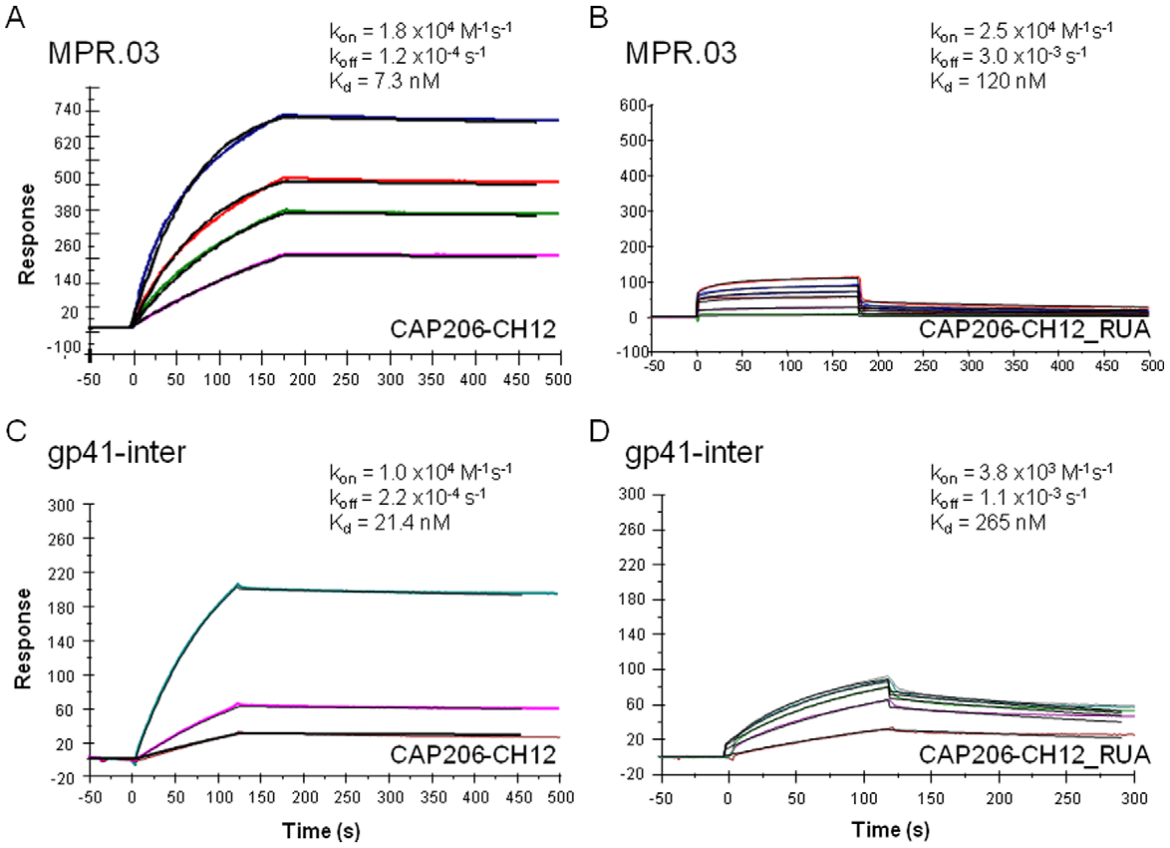
Binding of CAP206-CH12 mAb and CAP206-CH12 RUA to gp41 MPER peptide and gp41-inter protein. (A) Surface plasmon resonance binding Kd and rate constants for CAP206-CH12 and (B) CAP206-CH12_RUA to MPR.03 peptide. Biotinylated MPER peptide was anchored to a streptavidin coated chip and antibodies were injected at varying concentrations (40 – 5 ug/mL and 100 – 20 ug/mL for CAP206-CH12 mAb and RUA respectively) over the peptide surfaces. Binding of (C) CAP206-CH12 and (D) CAP206-CH12_RUA to gp41-inter protein showed that the RUA Kd was an order of magnitude weaker. For gp41-inter binding, each mAb was captured to about 1000 RU on an anti-Fc antibody immobilized surface (24). Varying concentrations (10–100 ug/mL) of gp41-inter protein was injected over the antibody captured surfaces. Data is representative of two independent measurements and in a second experiment, the measured binding Kd was 15.1 nM and 137 nM for CAP-206-CH12 mAb and RUA respectively.

In surface plasmon resonance measurements, CAP206-CH12 mAb bound to MPR.03 peptide with a K_d_ of 7.3 nM (Figure 2A) which was comparable to that of 4E10. Alanine scanning studies showed that the CAP206-CH12 binding epitope spanned the _672_WF(N/D)IT_676_ motif, which overlapped with the 4E10 [9] (Figure S2) and Z13e1 epitopes [12]. With the exception of T676A (∼30% reduced), all other substitutions reduced CAP206-CH12 binding by >50% relative to the wild type peptide. Although CAP206-CH12 epitope included two critical residues of the 4E10 epitope, W_672_ and F_673_ [9], single alanine substitution of either W_672_ or F_673_ had a more drastic effect on 4E10 binding (<20% binding) than on CAP206-CH12 (30–40% binding) (Figure S2). A critical residue for Z13e1 binding and neutralization, N_671_ and residues N-terminus to this (S_668_LW_670_), did not play a role in CAP206-CH12 binding (Figure S2). Thus, the core epitope of CAP206-CH12 was slightly narrower and included C-terminus residues of gp41 MPER that overlapped with the epitope of 4E10.

Previously, 2F5 and 4E10 mAbs were shown to bind strongly with exceptionally slow off-rates to trimeric 92UG gp41-inter, a protein that mimics the pre-hairpin intermediate state of gp41 [18]. CAP206-CH12 bound to gp41-inter suggesting that it recognizes the MPER presented in the pre-hairpin conformation of gp41 (Figure 2C). However, when compared to 4E10 binding (K_d_ = 1.6 nM; k_off_ = 1.5×10^−5^ s^−1^, 25), CAP206-CH12 binding to gp41-inter was considerably weaker (K_d_ = 21.4 nM) and displayed about 10-fold faster off-rates (k_off_ = 2.2×10^−4^ s^−1^). This may explain the lower neutralization potency of CAP206-CH12 when compared to that of 4E10.

The putative CAP206-CH12 germline antibody or reverted unmutated ancestor, CAP206-CH12_RUA also bound to MPR.03 peptide but with a binding K_d_ of 120 nM (Figure 2B), which was about 15-fold weaker than that of CAP206-CH12 mAb binding. CAP206-CH12_RUA also bound to gp41-inter but with an even weaker K_d_ of 265 nM and k_off_ (k_off_ = 1.1×10^−3^ s−1) which was about 20-fold faster than that of the mature CAP206-CH12 mAb (Figure 2D).

**Figure 3:**
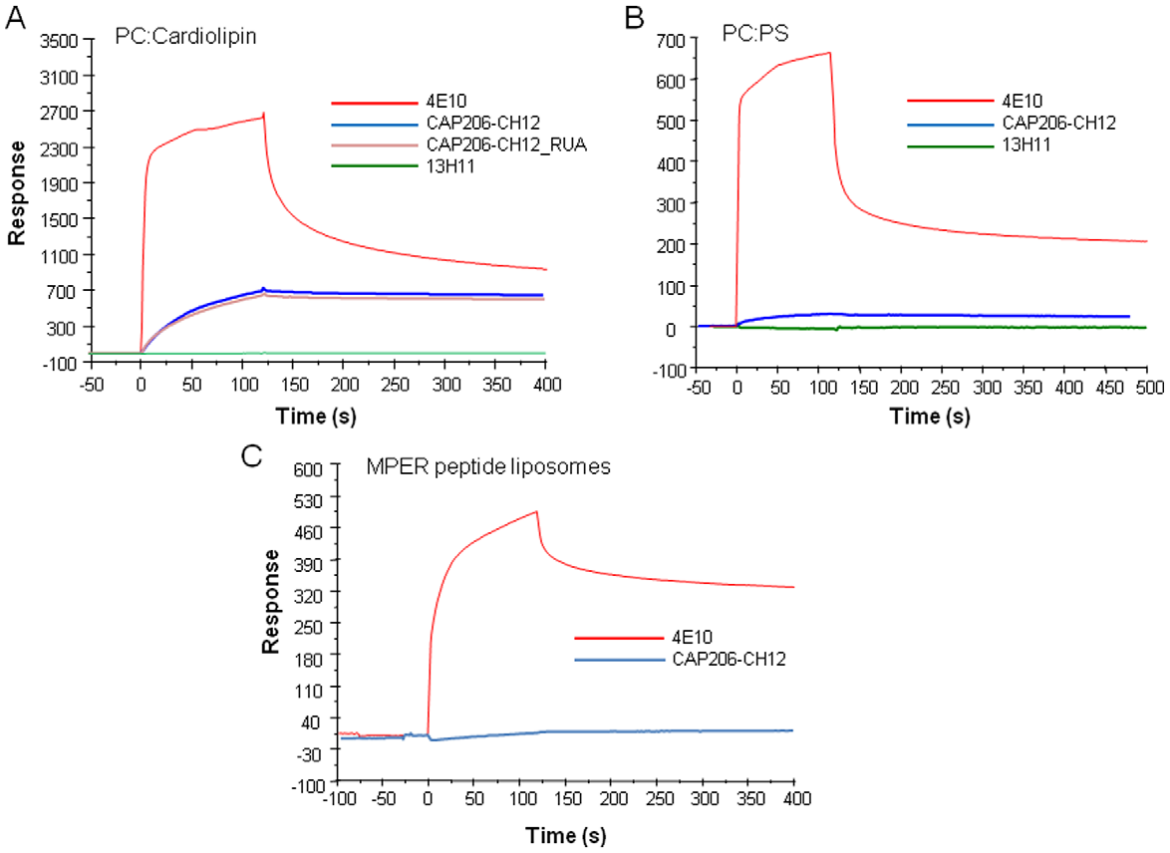
Binding of CAP206-CH12 with phospholipids and MPER peptide-liposomes. (A) CAP206-CH12 mAb bound weakly to both cardiolipin (PC∶CL 25∶75) and (B) phosphatidylserine (PC∶PS 25∶75) liposomes. Binding of 4E10 mAb to both forms of lipids and the lack of binding of the non-neutralizing gp41 MPER mAb 13H11 [40], as positive and negative controls respectively is also shown. The reactivity of CAP206-CH12_RUA to cardiolipin was similar to that of CAP206-CH12 mAb binding. (C) CAP206-CH12 mAb did not bind to MPER peptide liposomes (using MPER656a-GTHI) to which 4E10 mAb bound strongly. Binding of each mAb at 100 ug/mL to liposomes anchored on a hydrophobic L1 sensor chip was performed as described earlier [19], [40]. Data is representative of at least two independent measurements.

In contrast to 4E10, CAP206-CH12 and CAP206-CH12_RUA binding to cardiolipin or PS containing liposomes was considerably weaker (Figure 3A and 3B). Furthermore, CAP206-CH12 failed to bind to MPER656 peptide liposome complexes (Figure 3C). Since CAP206-CH12 bound to the same peptide (MPER656) in the absence of lipids (Figure 1B), the lack of binding of CAP206-CH12 to MPER peptide liposome complexes likely reflects either its inability to interact with membrane embedded critical residues or with the conformation of the MPER peptide on liposomes with the net result that CAP206-CH12 mAb cannot extract the MPER from lipid [19], [20].

**Figure 4:**
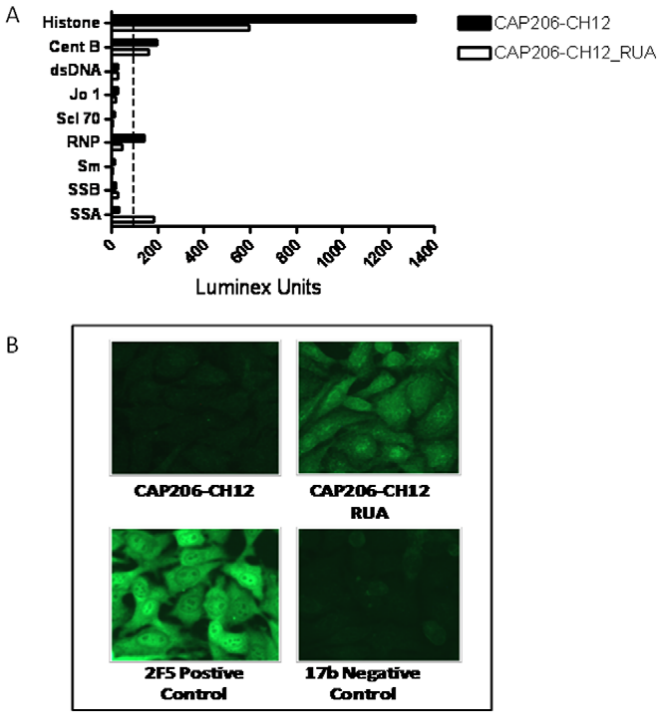
Polyspecificity of CAP206-CH12 and its RUA. (A) Reactivity of both CAP206-CH12 and CAP206-CH12_RUA with autoantigens. Both antibodies reacted with Histone and Centromere B. CAP206-CH12 also reacted with RNP (Ribonucleoprotein), while CAP206-CH12_RUA reacted with SSA (Ro/Sjogren syndrome autoantigen). Other antigens tested but were not-reactive were dsDNA (double stranded DNA), Jo 1 (histidyl-tRNA synthetase), Scl 70 (Scleroderma), Sm (Smith antigen) and SSB (Sjogren syndrome antigen B). Experiment shown is representative of three. (B) CAP206-CH12 did not react with human HEp-2 epithelial cells while CAP206-CH12_RUA showed nuclear and cytoplasmic reactivity (top panels). The 2F5 mAb reacted with a diffuse cytoplasmic and nuclear pattern as previously reported [10] while 17b (gp120 CD4i mAb) used as a negative control showed no reactivity (bottom panels). Each antibody was used at 50 ug/mL and each image is at ×400 magnification. Data is representative of three individual experiments.

Like mAb 4E10, CAP206-CH12 was markedly polyreactive and bound to histones, centromere B autoantigens and ribonucleoprotein (Figure 4A). The RUA also reacted with SSA. In the Hep-2 cell fluorescence assay, CAP206-CH12 did not react with Hep-2 epithelial cells while its RUA reacted in a nuclear and cytoplasmic pattern (Figure 4B).

### Neutralizing Activity of CAP206-CH12

The functional activity of mAb CAP206-CH12 was tested in the TZM-bl pseudovirus neutralization assay using viruses against which the CAP206 plasma was active. Of the 6 viruses tested, 4 were shown to be sensitive to mAb CAP206-CH12 (Table 2). This included the autologous virus as well as 2 subtype C and 1 subtype B virus. CAP206-CH12 when tested at 32 µg/ml did not neutralize 2 other viruses against which the plasma showed low levels of activity. Comparison of the IC_50_ values suggested that CAP206-CH12 was similar in potency to the mAb Z13e1 and consistent with earlier data using polyclonal antibodies eluted from MPR.03 peptides [15]. CAP206-CH12 was considerably less potent than mAb 4E10, but more cross-reactive than 2F5 which generally fails to neutralize subtype C viruses [21]. When tested against a large unselected panel of primary Tier 2 viruses of subtypes A, B and C, CAP206-CH12 neutralized 2 of the 26 heterologous viruses; Du156.12 and ZM197M.PB7 previously shown to be neutralized by this mAb, in addition to the autologous virus (Table S1).

**Table 2:** CAP206-CH12 mAb neutralization of viruses sensitive to CAP206 plasma.

Pseudovirus	Subtype	ID_50_	IC_50_ (μg/ml)			
		CAP206 plasma	CAP206-CH12	Z13e1	2F5	4E10
CAP206.B5*	C	6,143	**5.9**	nd	>25	0.1
ZM197M.PB7	C	256	**13**	30	>25	1.1
Du156.12	C	232	**14.9**	4.7	>25	0.2
TRO.11	B	212	**17.5**	13.3	>25	0.3
QHO692.42	B	125	>32	46	1.81	6.5
Du422.1	C	90	>32	nd	>25	0.3

Values are either the reciprocal plasma dilution (ID_50_) or mAb concentration (IC_50_, μg/ml) at which relative luminescence units (RLUs) were reduced 50% in TZM-bl cells compared to virus control wells (no test sample).

*indicates the autologous transmitted/founder virus.

Interestingly when a subset of these viruses was tested using TZM-bl cells in which the FcRγI receptor had been transfected, increased potency and breadth of CAP206-CH12 was observed as has been previously reported for mAb 4E10 (Table 3) [22]. Thus, there was a 2–12 fold increase in sensitivity and two viruses (Du422.1 and SC422661.8) that were previously resistant were now sensitive to CAP206-CH12.

**Table 3:** Enhancement of CAP206-CH12 neutralization in TZM-bl cells expressing FcRγ1.

Pseudovirus	Subtype	IC_50_ (μg/ml)		
		CAP206-CH12	2F5	4E10
ZM197M.PB7	C	0.3	0.06	<0.01
SC422661.8	B	0.4	<0.01	<0.01
Du156.12	C	0.6	>25	<0.01
CAP206.B5*	C	0.7	>25	<0.01
Du422.1	C	2.7	>25	<0.01
QH0692.42	B	>32	<0.01	0.11

Values are mAb concentration (IC_50_, μg/ml) at which relative luminescence units (RLUs) were reduced 50% compared to virus control wells (no test sample).

*indicates the autologous transmitted/founder virus.

### Mapping of MPER Residues Important for Neutralization

We used the subtype C pseudovirus, COT6.15 which has been alanine-scanned across the MPER, to assess which residues were crucial for neutralization. As shown in Table 4, the mutations D674A, N677A and W680A had a major effect on neutralization by CAP206-CH12 while F673A had a lesser effect. Two of these residues (F_673_ and W_680_) are also crucial for 4E10 neutralization and both F_673_ and D_674_ were previously shown to affect neutralization by CAP206 plasma antibodies [15].

**Table 4:** MPER amino acid residues critical for CAP206-CH12 neutralization.

COT6.15	CAP206-CH12 mAb		CAP206 plasma		4E10 mAb	
MPER mutants	IC_50_	ratio to WT	ID_50_	ratio to WT	IC_50_	ratio to WT
Wild-Type (WT)	11.0	1.0	1,256	1.0	0.9	1.0
W670A	4.1	0.4	1,054	1.2	0.1	0.1
S671A	2.6	0.2	1,614	0.8	0.0	0.0
W672A	1.1	0.1	2,244	0.6	**>25**	**>25**
F673A	**23**	**2.1**	**498**	**2.5**	**>25**	**>25**
D674A	**150**	**13.7**	**<50**	**>25**	1.4	1.6
I675A	14.6	1.3	2,065	0.6	0.0	0.0
T676A	15.4	1.4	895	1.4	**21.8**	**24.2**
K677A	**150**	**13.7**	2,151	0.6	0.1	0.1
W678A	1.8	0.2	1,885	0.7	0.1	0.1
L679A	1.7	0.2	1,448	0.9	0.1	0.1
W680A	**150**	**13.7**	904	1.4	**10.9**	**12.1**

Values are either the mAb concentration (IC_50_, μg/ml) or reciprocal plasma dilution (ID_50_) at which relative luminescence units (RLUs) were reduced 50% compared to wild-type COT6.15 virus.

Analysis of MPER sequences of viruses sensitive to CAP206-CH12 showed that all had an aspartic acid at position 674 similar to the sequence present in the MPR.03 peptide (Figure S3). The amino acid at position 677, the other site identified by alanine substitution mapping as important for CAP206-CH12 neutralization, was more variable with sensitive isolates tolerating K, N or H. QH0692.42 was sensitive to plasma antibodies but not to CAP206-CH12 and had the nominal D_674_ but had an arginine at position 677 possibly accounting for its lack of sensitivity. Other isolates that had D_674_ and either K or N at 677 were resistant suggesting that simply having the nominal epitope was not sufficient and other aspects such as exposure of the MPER are likely important in determining CAP206-CH12 sensitivity. The 2 other residues F_673_ and W_680_ identified in the COT6.15 scanning as important for neutralization (Table 4) were highly conserved across all sensitive and resistance isolates. Sites shown to be important for binding (W_672_, I_675_ and T_676_) (Figure S2) were also either highly conserved or did not distinguish between the sensitive and resistant isolates.

## Discussion

In this study we demonstrate the power of prior epitope mapping of plasma neutralizing antibodies that allowed for the rationale design of an antigen-specific memory B cell receptor ligand (bait). Furthermore, the use of single cell sorting with dual-labeled ligands facilitated the efficient and specific selection of relevant memory B cells. Remarkably, we saw utilization of the same V_H_ and V_L_ families by this new MPER neutralizing mAb, CAP206-CH12, as that used by the prototype MPER mAb 4E10. Overall, this approach has significant potential for the discovery of additional novel neutralizing anti-MPER mAbs from HIV-1 infected individuals, which will allow for further interrogation of this important gp41 vaccine target.

While the CAP206-CH12 mAb neutralized approximately two-thirds of the viruses sensitive to CAP206 plasma antibodies, this mAb did not recapitulate all the plasma neutralizing activity. This suggests that this type of antibody was responsible for a portion of the breadth observed in plasma, and that full coverage would likely be provided by additional mAbs, possibly somatic variants of CAP206-CH12. Indeed the isolation of additional V_H_1-69 heavy chains from this individual supported this notion, although this could not be proven due to the inability to express them as functional mAbs. Nonetheless, the CAP206-CH12 mAb epitope directly overlapped the epitope of plasma antibodies confirming that it comprised a component of plasma neutralizing activity. Overall, CAP206-CH12 showed modest neutralization activity and limited potency, similar to Z13e1, and reacted with both subtype C and B viruses. While the CAP206-CH12 mAb was polyreactive, unlike 2F5 and 4E10, CAP206-CH12 did not bind lipids avidly. Since both 2F5 and 4E10 rely on lipid reactivity for virion membrane binding in order to mediate neutralization [19], [23], [24], one hypothesis is that the neutralization potency of CAP206-CH12 may be limited by its minimal lipid reactivity which is under investigation. However, neutralization breadth and potency of CAP206-CH12 could be enhanced by engaging with FcRγΙ receptors, similar to 4E10 and 2F5. This is a feature unique to anti-MPER mAbs and may be related to the pre-positioning of these mAbs during the transient fusion process [22].

It was striking that CAP206-CH12 utilized the V_H_1-69 and Vκ3-20 similar to the gp41 antibody, 4E10. Gorny and colleagues have previously reported that non-neutralizing human antibodies that bind to epitopes in the cluster II region of gp41 (N-terminal to the MPER) frequently use a V_H_1-69 Ig heavy chain [25]. Other gp41 antibodies, such as D5 [26] and HK20 that binds to the stalk of gp41 [27] also utilize V_H_1-69. Another example of restricted usage of V_H_1-69 has recently been reported following the isolation of influenza broadly neutralizing antibodies to the stalk of hemagglutinin [28]. V_H_1-69 antibodies are hydrophobic and one hypothesis is that these antibodies are preferentially used for regions of virus envelopes that are in close proximity to viral membranes. Alternatively, Johnson and co-workers reported that the percentage of the blood B cell repertoire that use V_H_1-69 is directly related to the V_H_1-69 copy number [29]. Thus, both host and immunogen factors may give rise to preferential usage of V_H_1-69 in anti-viral responses.

Another interesting finding was that both CAP206-CH12 and another anti-MPER mAb, Z13e1 had the YYYYMD motif in their CDRH3 as a result of a shared J allele. In the case of Z13e1, three of the Tyr residues positioned at the base of CDRH3 make contacts with the peptide [12] and thus CAP206-CH12 could potentially utilize these Tyr residues in a similar manner. It is notable that both 4E10 and Z13e1 have a flexible CDRH3 tip that bends away from the bound antigen [12]. While the 4E10 CDRH3 apex is involved in both lipid binding and neutralization [11], the flexibility of the Z13e1 CDRH3 tip could allow it to engage the membrane–bound epitope [12]. CAP206-CH12, has a slightly shorter CDRH3 and includes some flexible residues adjacent to the Tyr motif but lacks hydrophobic residues W or F, that are present in both the 4E10 and Z13e1 CDRH3 apex (4E10 – GWGWLG; Z13e1 – SGFLN). Since CAP206-CH12 did not bind to MPER peptide liposomes, in which MPER C-terminus hydrophobic residues are membrane immersed [19], it is likely that CAP206-CH12 targets a different gp41 conformation, possibly one in which the MPER is more solvent exposed.

The epitope of the CAP206-CH12 mAb overlapped that of both 4E10 and Z13e1. The Z13e1 epitope spans residues S_668_LWNWFDITN_677_ [30] while binding studies identified the epitope of CAP206-CH12 to WF(N/D)IT, which does not include residues N-terminus to W_670_. In particular, position 674 was shown to be critical for both Z13e1 and CAP206-CH12. Residue F673, a major determinant of the 4E10 epitope was also important for the CAP206-CH12 mAb as shown in both binding and neutralization studies. For MPER mAbs that bind to overlapping residues, differences in both orientation and conformation of gp41 recognized by 4E10 and Z13e1 have been described [12], [31]. Based on the mapping and neutralization mutagenesis data, it is likely that CAP206-CH12 binds to a 4E10-favored W_672_/F_673_ accessible MPER conformation.

The lack of binding to denatured or plate bound JR-FL or ConS gp140 and also to gp41 proteins suggests that the epitope recognized by CAP206-CH12 is conformation sensitive. Even in cases where the MPER sequences were identical, differences elsewhere in gp41 and also within gp120 might affect gp120-gp41 interactions and the overall conformation of MPER accounting for the lack of binding. Furthermore, while denaturing gp140 proteins would expose the linear sequence, it does not preserve the secondary structure which may account for some of the differences seen between peptides and denatured proteins. However, like the previously described broadly neutralizing gp41 MPER antibodies (2F5 and 4E10), CAP206-CH12 bound to gp41-inter protein. Thus CAP206-CH12 is capable of targeting the pre-hairpin intermediate conformation of gp41, which is not recognized by non-neutralizing gp41 cluster II antibodies [18], [32]. However, the relatively weaker binding K_d_ of CAP206-CH12 for gp41-inter protein is consistent with its poorer neutralization activity. Both lipid reactivity and a stable binding of MPER antibodies with extremely slow off-rates have been proposed to be a requirement for the capture of the transient pre-hairpin intermediate conformation and subsequently the blocking of cell fusion [11], [18]. CAP206-CH12 mAb, with its weaker reactivity and relatively faster off-rates will therefore, be less efficient in capturing the transient intermediate state of gp41. The even weaker binding of CAP206-CH12_RUA to gp41-inter suggests that enhancement in gp41-inter binding is acquired during maturation of the antibody.

The finding that the germline-like antibody of CAP206-CH12 bound the MPR.03 peptide and gp41-inter weakly allows us to propose a model whereby MPER epitopes on the early transmitted founder virions initiated the hypermutation process in a naive B cell during acute HIV-1 infection. The fact that the first binding antibodies in HIV-1 infection are to gp41 [33], suggests that CAP206-CH12 may be derived from one of the earliest B cell precursors that encountered HIV. Initially these antibodies were non-neutralizing but through a process of affinity maturation acquired limited neutralization capacity. The polyreactivity of the germline antibody may also have played a role in its maturation which may be a common feature of antibodies targeting gp41 [34], [35]. The mutation rate of the heavy chain of CAP206-CH12 while lower than that of other MPER neutralizing mAbs was roughly similar to what is seen among other broadly neutralizing mAbs [36], [37]. It is possible that CAP206-CH12 represents an intermediate in the maturation process of this clonotype. Further work aims to examine the ontogeny of this antibody specificity to explore this hypothesis.

Finally, our studies show that epitope mapping of plasma antibodies followed by the rational design of fluoresceinated MPER peptide tetramers can successfully isolate antigen-reactive single B cells for Ig rescue. Scheid and colleagues have previously used fluoresceinated gp140 envelope for this purpose for isolation of Env-reactive B cells [5] and more recently VRC01 a potent anti-CD4bs mAb was isolated using a modified gp120 protein [6]. Our strategy combined an antigen-specific probe with two color labeling to increase sensitivity and enhance the specificity of isolated antibodies. The methods used here should allow for the isolation of broadly neutralizing antibodies from many subjects with neutralizing antibody breadth where suitable antigens can be designed. Study of the B cells and their reverted unmutated ancestors should prove useful in design of immunogens capable of activating B cell receptors of naïve cells that are able to develop into anti-HIV-1 antibodies with neutralizing breadth.

## Materials and Methods

### Human Samples and Ethics Statement

Stored plasma and PBMC from CAP206 an HIV-1 subtype C chronically infected individual were used for this study. This participant was part of the CAPRISA 002 Acute infection cohort whose antibody neutralization profile has been studied since the point of seroconversion [13], [38]. This study was approved by the IRB's of the Universities of KwaZulu Natal and Witwatersrand in South Africa and Duke University. Written informed consent was obtained from all study participants.

### Reagents

The MPR.03 biotinylated peptide containing lysines at both ends for solubility (KKKNEQELLELDKWASLWNWFDITNWLWYIRKKK-biotin) and a scrambled version were purchased from CPC Scientific Inc (San Jose, CA). Peptides were >98% pure as tested by HPLC. MPER656 (Biotin-NEQELLELDKWASLWNWFNITNWLW), SP62 (2F5 peptide), SP400 (peptide from gp41 immunodominant region) and 4E10 peptides have been described previously [39]. The MPER656a-GTH1 peptide (NEQELLELDKWASLWNWFNITNWLWYIK-YKRWIILGLNKIVRMYS) was used in the preparation of MPER peptide liposomes [19], [40]. Proteins ConS gp140, JR-FL gp140 were generated as described [41] and MN gp41 was purchased from Immunodiagnostics, Woburn, MA. 4E10 and 2F5 mAbs were obtained from Polymun, Vienna, Austria. The CAP206.B5 virus was cloned by SGA from a pre-seroconversion plasma sample and is the autologous transmitted founder virus. A gp140 protein, CAP206.B5.140C with the gp120-gp41 cleavage site mutated (REKR motif), was used in Western blots and ELISA's. Other viruses were from the standard clade A, B and C reference panels [42], [43], [44]. MPER mutants made in the subtype C virus, COT6.15 have been described previously [15]. HIVIG-C is a pool of purified IgG from five blood bank donors in Johannesburg, South Africa who were confirmed by sequence analysis to be infected with subtype C HIV-1.

### Preparation of Tetramers

Tetramers were prepared using the biotinylated MPR.03 peptide with both allophycocyanin (APC) and Pacific Blue labelled streptavidins and titered on antibody-coated beads and on antibody expressing cell lines (using the 13H11 and 2F5 mAbs which both bind the MPR.03 peptide). Briefly, excess biotinylated peptide (approximately 33∶1 molar ratio of peptide to streptavidin for fluorochrome-labeled tetramers) was incubated at 4°C overnight and isolated using gel filtration on Micro BioSpin 30 columns. Tetramers were assayed for final concentration determined using standard spectrophotometric techniques. Final titers were determined using a combination of 2F5-coated beads and 13H11- expressing cell lines. Tetramers were used in equimolar amounts in combination with a panel of mAbs to identify memory B cells in PBMC.

### Staining and Sorting B Cell Populations

Thawed PBMC were stained with a combination of the following antibodies: CD3 PE-Cy5, CD14 PE-Cy5, CD16 PE-Cy5, CD235a PE-Cy5, CD19 APC-Cy7, CD27 PE-Cy7, CD38 APC-Cy5.5 and IgG-PE (BD Biosciences, Mountain View, CA and Invitrogen, Carlsbad, CA). All antibodies were titered and used at optimal concentrations for flow cytometry. Memory B cells were gated as CD3^neg^, CD14^neg^, CD16^neg^, CD235a^neg^, CD19^pos^, CD27^hi^, CD38l^ow^ and IgG^+^. Tetramer-stained B cells were sorted as single cells into wells of a 96-well plate, selecting those cells that were labelled by both tetramers. Cells were stored in reverse-transcriptase reaction buffer at −80°C until use [45]. Flow cytometric data was acquired on a BD FACS Aria and the data analyzed using FlowJo.

### Isolation of Ig Variable Gene Transcripts

The genes encoding V_H_ and V_L_ were amplified by PCR using a modification of the method described by Tiller and co-workers [46]. Briefly RNA from single sorted cells was reverse transcribed using Superscript III in the presence of primers specific for human IgG, IgM, IgD, IgA1, IgA2, kappa and lambda constant gene regions [45]. The V_H_, V_K_ and V_L_ genes were amplified from this cDNA separately in a 96-well nested PCR as described and analysed on 2% agarose gels [45]. The second round PCR included tagged primer sequences at the 5′ end which permitted assembling of the V_H_ and V_L_ genes into functional linear Ig gene expression cassettes (see below). PCR products were purified and sequenced. The variable gene segments and the potential functionality of the Ig genes was determined using the SoDA program [47].

### Expression of Recombinant Antibodies from Linear Expression Cassettes

Three linear Ig expression cassettes each containing the CMV promoter and human Ig leader as one fragment were used for small-scale expression and specificity analysis [45]. Fragments for the heavy and light chains comprised either the IgG1 constant region, Ig kappa constant region or Ig lambda constant region attached to poly A signal sequences. These two fragments plus either V_H_, V_K_ or V_L_ genes amplified from single B cells were assembled by overlapping PCR. PCR products containing linear full-length Ig heavy- and light-chain genes were purified and the paired Ig heavy and light-chain products co-transfected into 293T cells grown in 12-well plates using Fugene. Cultures were fed 6–12 hrs later with ∼2 mls fresh medium containing 2% FCS and incubated for 72 hours at 37°C in a 5% CO_2_ incubator. Thereafter culture supernatants were harvested for antibody characterization.

### Design and Synthesis of CAP206-CH12 Inferred Reverted Unmutated Ancestor

The SoDA program [47] was used to infer the reverted unmutated ancestor (RUA) of CAP206-CH12. These genes were synthesized (GeneScript, Piscataway, NJ) and cloned as full-length IgG1 for heavy chain and full-length kappa light chain genes into pcDNA3.1 plasmid (Invitrogen; Carlsbad, CA) using standard recombinant techniques.

### Production of Purified Recombinant MAbs

The selected Ig V_H_ and V_K_ genes from CAP206-CH12 were cloned into human Igγ and Igκ expression vectors in pcDNA3.3 [45]. Clones with the correct size inserts were sequenced to confirm identity with the original PCR products. For production of purified antibodies of CAP206-CH12 and CAP206-CH12_RUA by batch transient transfections, 10–20 T-175 flasks of 293T cells grown at 80–90% confluency in serum-free media were co-transfected with plasmids expressing HIV-1 specific Ig heavy- and light chain genes using Fugene (Qiagen, Valencia, CA). Recombinant antibodies were purified using anti-human IgG heavy-chain specific antibody-agarose columns.

### ELISA and Western blots

Supernatants from the small scale transfections and purified mAbs were tested for reactivity using various peptides and proteins in an ELISA as described [45]. An anti-cardiolipin ELISA was used as previously described [48], [49]. Autoantibodies were measured by the FDA-approved AtheNA Multi-Lyte® ANA II Test Kit from Zeus Scientific, Inc. per the manufacturer's instructions and as described previously [10]. Western blots were performed using NuPAGE® Novex 4–12% Bis-Tris gels, 1.0 µg per lane for coomassie blue stain and 0.5 µg per lane for Western blot. Gels were run at 200 V for 50 min under non-reducing and reducing conditions. The coomassie images were acquired in Odyssey Infrared Imaging System (Li Cor Bioscience, Lincoln, NE). Alkaline-phosphatase conjugated goat anti-human IgG (Sigma, St. Louis, MO) at 1∶5000 dilution was applied as secondary Ab. The membranes were developed in Western Blue® Stabilized Substrate (Promega, Madison, WI).

### Surface Plasmon Resonance

MPER656, MPR.03 and a scrambled version of MPR.03 were individually anchored on a BIAcore SA sensor chip as described previously [40], [50]. Binding assays were performed on a BIAcore 3000 instrument at 25°C and data analyzed using the BIAevaluation 4.1 software (BIAcore) [40]. Peptides were injected until 100–150 response units of binding to streptavidin were observed. Trimeric 92UG gp41-inter protein was produced and purified as described earlier [18]. Antibodies were captured on an anti-human IgFc immobilized surface [40] and varying concentrations of gp41-inter protein was injected over the antibody surfaces. Binding K_d_ measurements were made as described earlier [18], [19], [39], [40].

### Neutralization Assays

The TZM-bl pseudovirus assay was used to assess the neutralization activity of CAP206-CH12 and control mAbs against viruses that were sensitive to CAP206 plasma antibodies as well as to a large panel of 26 unselected heterologous Tier 2 viruses from multiple subtypes available in Dr David Montefiori's CAVD Consortium. Mapping of residues in the MPER crucial for CAP206-CH12 neutralization made use of the COT6.15 virus which was alanine-scanned across the MPER. The mAb concentration at which 50% of virus neutralization was seen (IC_50_ value) was reported. Purified mAb was used for these experiments to avoid interference from transfection reagents. Plasma was heat inactivated prior to performing neutralization assays.

## Supporting Information

Figure S1 **Amino acid alignment of CAP206-CH12 V_H_ and V_L_ chains with 4E10 and Z13e1 (A) and nucleotide sequences of CAP206-CH12 and CAP206-CH12_RUA heavy and light chains (B).**
(PPTX)

Figure S2 **Mapping of a MPER amino acid residues important for CAP206-CH12 and 4E10 binding.** Bar graphs show relative binding of CAP206-CH12 and 4E10 mAbs to alanine scanned MPER656 peptides. Residues highlighted in red show more than 50% reduction in binding relative to wild-type peptide. Individual SPR curves for 3 residues W_672_, F_673_, N_674_ (boxed in bar graph) normalized to 100% binding show the differential effect of the substitution on 4E10 (blue) and CAP206-CH12 (red) binding. While each of the substitutions drastically reduced the binding (>50%) of CAP206-CH12 relative to wild-type, the substitutions show marked differences in the off-rates of the binding of 4E10 to W672A and F673A peptides but not to N674A. Thus W672A and F673A substitutions had a more drastic effect on 4E10 binding than CAP206-CH12 binding. Alanine substituted mutants were designed by substituting single amino acids within the MPER peptide with the following sequence – Biotin-GGG-QELLELDKWASLWNWFNITNWLWYIK (MPER26). Relative binding of the mutants was measured using the wild type MPER656 peptide (Biotin-NEQELLELDKWASLWNWFNITNWLW). The binding of CAP206-CH12 mAb to all three MPER peptides (MPR.03, MPER656, MPER26) used in this study were similar. CAP206-CH12 bound to MPER26 and MPER656 peptide with Kd of 4.95 and 5.15 nM respectively.
(PDF)

Figure S3 **MPER sequences of viruses sensitive and resistant to CAP206-CH12 mAb.** Amino acids at positions 674 and 677 are highlighted in red. Residues at 673 and 680 (boxed) were conserved in all sensitive and resistant isolates.
(PDF)

Table S1 **Neutralization sensitivity of viruses to CAP206-CH12.**
(PDF)
