# Supplementary figures and images for: Isolation of a Human Anti-HIV gp41 Membrane Proximal Region Neutralizing Antibody by Antigen-Specific Single B Cell Sorting
Source: PLoS One. 2011 Sep 30;6(9):e23532. doi: 10.1371/journal.pone.0023532 (PMC3184076; doi:10.1371/journal.pone.0023532)

SOM Figure 2

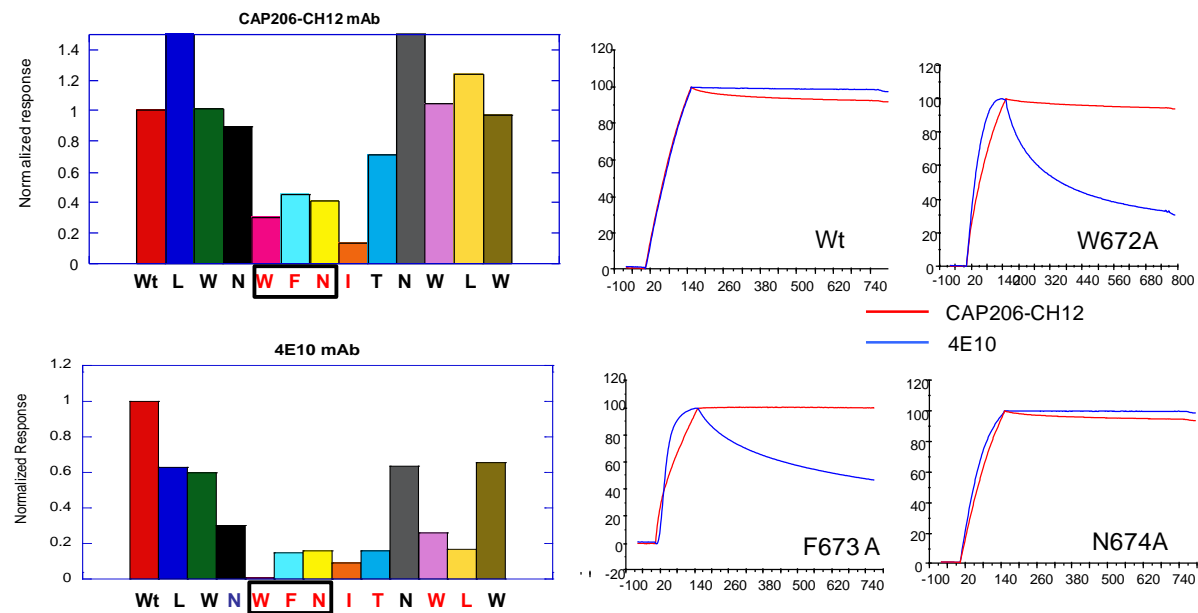

Supplement: Figure S2 — Mapping of a MPER amino acid residues important for CAP206-CH12 and 4E10 binding. Bar graphs show relative binding of CAP206-CH12 and 4E10 mAbs to alanine scanned MPER656 peptides. Residues highlighted in red show more than 50% reduction in binding relative to wild-type peptide. Individual SPR curves for 3 residues W672, F673, N674 (boxed in bar graph) normalized to 100% binding show the differential effect of the substitution on 4E10 (blue) and CAP206-CH12 (red) binding. While each of the substitutions drastically reduced the binding (>50%) of CAP206-CH12 relative to wild-type, the substitutions show marked differences in the off-rates of the binding of 4E10 to W672A and F673A peptides but not to N674A. Thus W672A and F673A substitutions had a more drastic effect on 4E10 binding than CAP206-CH12 binding. Alanine substituted mutants were designed by substituting single amino acids within the MPER peptide with the following sequence – Biotin-GGG-QELLELDKWASLWNWFNITNWLWYIK (MPER26). Relative binding of the mutants was measured using the wild type MPER656 peptide (Biotin-NEQELLELDKWASLWNWFNITNWLW). The binding of CAP206-CH12 mAb to all three MPER peptides (MPR.03, MPER656, MPER26) used in this study were similar. CAP206-CH12 bound to MPER26 and MPER656 peptide with Kd of 4.95 and 5.15 nM respectively. (PDF) [file pone.0023532.s002.pdf]
