# Supplementary material for: Isolation of a Human Anti-HIV gp41 Membrane Proximal Region Neutralizing Antibody by Antigen-Specific Single B Cell Sorting
Source: PLoS One. 2011 Sep 30;6(9):e23532. doi: 10.1371/journal.pone.0023532 (PMC3184076; doi:10.1371/journal.pone.0023532)
Supplement: Figure S3 — MPER sequences of viruses sensitive and resistant to CAP206-CH12 mAb. Amino acids at positions 674 and 677 are highlighted in red. Residues at 673 and 680 (boxed) were conserved in all sensitive and resistant isolates. (PDF) [file pone.0023532.s003.pdf]

SOM Figure 3

Sensitive

CAP206 8	KDLLALDSWKNLWNWFDITKWLWYIKIFII
ZM197M.PB7	KDLLALDKWNSLWSWFDITKWLWYIKIFIM
Du156.12	KDLLALDRWQNLWNWFDITNWLWYIKIFIM
TRO.11	QELLELDSWASLWNWFDISKWLWYIKIFIM
Du422.1	KDLLALDSWKNLWNWFDITNWLWYIKIFIM
SC422661.8	QELLELDKWASLWNWFDITHWLWYIKIFIM
COT6.15	QELLALDSWKNLWSWFDITKWLWYIKIFIM

Resistant

QH0692.42	HELLELDKWASLWNWFDITRWLWYIKIFIM
ZM214M.PL15	EDLLALDKWDNLWNWFDISKWLWYIKIFIM
Q842.d12	QDLLALDKWANLWNWFDISNWLWYIKIFIM
Q168.a2	QDLLALDKWASLWNWFDISKWLWYIRIFIM
Q461.e2	QDLLALDKWANLWNWFDVSKWLWYIKIFIM
Q23.17	KELLELDKWANLWSWFDISNWLWYIKIFII
AC10.0.29	QELLALDKWANLWNWFNITEWLWYIKIFIM
Du172.17	KDLLALDSWESLWSWFNITNWLWYIRIFIM
CAP45.G3	KDLLALDSWNNLWNWFNITNWLWYIKIFIM
6535.3	LELLELDKWGSLWNWFSISNWLWYIRIFII
PVO.4	QDLLALDKWESLWNWFSITKWLWYIKIFIM
RHPA4259.7	QELLALDKWASLWSWFSITHWLWYIKMFIM
