# Supplementary material for: Isolation of a Human Anti-HIV gp41 Membrane Proximal Region Neutralizing Antibody by Antigen-Specific Single B Cell Sorting
Source: PLoS One. 2011 Sep 30;6(9):e23532. doi: 10.1371/journal.pone.0023532 (PMC3184076; doi:10.1371/journal.pone.0023532)
Supplement: Table S1 — Neutralization sensitivity of viruses to CAP206-CH12. (PDF) [file pone.0023532.s004.pdf]

SOM Table 1: Neutralization sensitivity of viruses to mAb CAP206-CH12

	ID$_{50}$ in TZM-bl cells[1]			
	Clade	CAP206-CH12	2F5	4E10
6535.3	B	>32.00	3.60	1.06
QH0692.42	B	>32.00	2.38	7.64
SC422661.8	B	>32.00	0.72	1.47
PVO.4	B	>32.00	>25.00	7.42
AC10.0.29	B	>32.00	0.35	0.25
RHPA4259.7	B	>32.00	4.86	0.42
WEAU-d15.410.787	B	>32.00	0.32	0.52
BB1006-11.C3.1601	B	>32.00	1.84	1.16
BB1054-07.TC4.1499	B	>32.00	>25.00	2.15
700010040.C9.4520	B	>32.00	15.25	23.22
Du156.12	C	18.46	>25.00	0.25
Du172.17	C	>32.00	>25.00	0.41
Du422.1	C	>32.00	>25.00	3.51
ZM197M.PB7	C	20.21	13.67	0.56
ZM214M.PL15	C	>32.00	>25.00	16.89
CAP45.2.00.G3	C	>32.00	>25.00	>25.00
CAP206.1.B5	C	5.53	>25.00	0.09
CenvFs2_Pt1086_B2	C	>32.00	>25.00	0.97
CenvFs4_Pt0393_C3	C	>32.00	>25.00	8.45
CenvFs2_Pt1176_A3	C	>32.00	>25.00	6.08
CenvFs4_Pt2010_F5	C	>32.00	23.06	>25.00
Q23.17	A	>32.00	5.08	6.35
Q842.d12	A	>32.00	10.66	11.56
Q168.a2	A	>32.00	2.08	2.20
Q259.d2.17	A	>32.00	16.69	22.83
Q461.e2	A	>32.00	9.88	6.98
Q769.d22	A	>32.00	2.98	3.88

[1]*Values are the concentration (μg/ml) at which relative luminescence units (RLUs) were reduced 50% compared to virus control wells (no test sample).*
